# Risk factors for food insecurity and association with prenatal care utilization among women who took opioids during pregnancy

**DOI:** 10.21203/rs.3.rs-3921909/v1

**Published:** 2024-03-25

**Authors:** Lindsay M. Parlberg, Jamie E. Newman, Stephanie Merhar, Brenda Poindexter, Sara DeMauro, Scott Lorch, Myriam Peralta-Carcelen, Deanne Wilson-Costello, Namasivayam Ambalavanan, Catherine Limperopoulos, Nicole Mack, Jonathan M. Davis, Michele Walsh, Carla M. Bann

**Affiliations:** RTI International; RTI International; Cincinnati Children’s Hospital Medical Center; Emory University; Children’s Hospital of Philadelphia; Children’s Hospital of Philadelphia; University of Alabama; Case Western Reserve University; University of Alabama; Children’s National Medical Center; RTI International; Tufts University; Eunice Kennedy Shriver National Institute of Child Health and Human Development; RTI International

**Keywords:** Food insecurity, housing instability, antenatal opioid exposure, social determinants of health

## Abstract

**Background.:**

Food insecurity during pregnancy is associated with poorer outcomes for both mothers and their newborns. Given the ongoing opioid crisis in the United States, mothers who take opioids during pregnancy may be at particular risk of experiencing food insecurity.

**Methods.:**

This research utilized data from 254 biological mothers of infants in the Advancing Clinical Trials in Neonatal Opioid Withdrawal Syndrome (ACT NOW) Outcomes of Babies with Opioid Exposure (OBOE) Study. We examined factors associated with food insecurity among mothers of infants with antenatal opioid exposure and their unexposed (control) counterparts. Chi-square tests and logistic regression were used to compare food insecurity by sociodemographic characteristics, opioid use, prior traumatic experiences, and housing instability. Similar analyses were conducted to examine the relationship between food insecurity during pregnancy and receipt of adequate prenatal care.

**Results.:**

Overall, 58 (23%) of the mothers screened positive for food insecurity. Food insecurity was more common among mothers who took opioids during pregnancy (28% vs. 14%; p =0.007), had public insurance (25% vs. 8%; p = 0.027), had housing instability (28% vs. 11%, p = 0.002), experienced three or more adverse experiences in their childhood (37% vs. 17%; p < 0.001), and reported physical or emotional abuse during their pregnancy (44% vs. 17%; p < 0.001). Mothers with food insecurity during pregnancy were less likely to have received adequate prenatal care (78% vs. 90%; p = 0.020). This difference remained after controlling for demographic characteristics (AOR (95% CI) = 0.39 (0.16, 1.00), p = 0.049).

**Conclusions.:**

This study adds to the body of evidence supporting the need for screening and development of interventions to address food insecurity during pregnancy, particularly among mothers of infants with antenatal opioid exposure, for which limited data are available. The findings revealed that food insecurity frequently co-occurs with housing instability and prior trauma, indicating that a multifaceted intervention incorporating principles of trauma-informed health care is needed. Although those with food insecurity are at increased risk for poor pregnancy outcomes, they were less likely to have received adequate prenatal care despite high levels of public insurance coverage among study participants, suggesting additional strategies are needed to address barriers to health care among this population.

**Trial registration.:**

The Outcomes of Babies with Opioid Exposure (OBOE) Study is registered at Clinical Trials.gov (NCT04149509) (04/11/2019).

## INTRODUCTION

Food and housing make up the most basic family needs yet food insecurity and housing instability are a present and growing problem with implications on an individual’s health and well-being [1, 2]. Food insecurity is a condition in which households have limited or uncertain access to adequate food. Food insecurity is related to poorer health outcomes in both children and adults, including adverse physical and cognitive outcomes for affected children [3]. Individuals and families who experience food insecurity are less likely to seek needed medical care and more likely to postpone medications and miss treatment appointments than their food secure counterparts [4]. Previous studies have suggested that infants whose mothers took opioids during pregnancy are more likely to require treatment for neonatal opioid withdrawal syndrome (NOWS) if their mothers also experienced food insecurity during pregnancy [3]. Similarly it has been documented that addressing interrelated social determinants of health, such as housing instability and food insecurity, is associated with improvements in population health and reductions in health care spending [5, 6].

Although a universal definition for housing instability does not exist, housing instability is recognized as a key social determinant of health that encompasses having trouble paying rent, overcrowding, moving frequently, staying with relatives, or spending the bulk of income on housing [7]. Throughout the United States, households are classified as cost burdened if they spend more than 30% of their income on housing and severely cost burdened if they spend more than 50% of their income on housing [8]. For many cost-burdened households there is little income left over each month to spend on necessities such as health care, food, clothing, and utilities [3].

The overwhelming consequences of the opioid epidemic in the United States for mothers and infants have grown as the rise of opioid use and misuse in pregnancy has mirrored that of the general population. During delivery hospitalization there were four times as many women with an opioid use disorder in 2014 compared with 1999 [9, 10]. Pregnant and postpartum women who use or misuse substances are at high risk for adverse maternal and infant outcomes, including preterm labor, and complications related to delivery, and often experience other challenges intensified by social determinants of health [11, 12]. These challenges may result in increased stress, mental health problems, and an increased risk of disease [13].

Given the importance of food and housing status as a determinant of health and the ongoing opioid crisis in the United States, families of infants with antenatal opioid exposure may be at particular risk of experiencing food insecurity and housing instability. Although the impacts of food insecurity and housing instability on health have been studied, there is a gap in our knowledge of factors associated with these key social determinants of health, specifically among mothers of infants with antenatal opioid exposure. Identifying such factors is essential to effectively recommend solutions for improved health within this population.

To better understand and assess these issues, we examined factors associated with housing instability and food insecurity among mothers of infants with antenatal opioid exposure and their unexposed (control) counterparts as part of a multisite prospective longitudinal cohort study. The goals of these analyses are to (1) compare prevalence of food insecurity among at-risk mothers who took opioids during pregnancy to those who did not take opioids; (2) investigate the relationship between food insecurity and housing and neighborhood characteristics; (3) examine whether food insecurity varies among demographic subgroups, including those with prior traumatic experiences; and (4) explore whether food insecurity is related to receiving adequate prenatal care. The study results could assist in defining priorities around screening for and implementing interventions to address these critical social determinants of health.

## METHODS

### Study Design

This research utilized data available from the Advancing Clinical Trials in Neonatal Opioid Withdrawal Syndrome (ACT NOW) Outcomes of Babies with Opioid Exposure (OBOE) Study, a multisite prospective longitudinal cohort study of the outcomes of infants with antenatal opioid exposure and controls from birth to 2 years of age (Clinical Trials.gov NCT04149509) (04/11/2019). The primary OBOE Study objectives are to determine the impact of antenatal opioid exposure on brain development and neurodevelopmental outcomes over the first 2 years of life and explore whether family, home, and community factors modify developmental trajectories during this critical time period. Data collection includes a series of direct assessment, parent/caregiver reports, and neuroimaging at regular intervals from birth to 2 years of age. Additional details on the study protocol, including assessments, are available elsewhere [14].

### Participants

All birthing mothers at each participating clinical site hospital were screened for OBOE Study eligibility. Mothers were included if their infants were born at or after 37 weeks gestation with second or third trimester opioid exposure as determined by maternal history; maternal urine toxicology screen at delivery; or infant urine, meconium, or umbilical cord toxicology screen. Exclusion criteria included heavy alcohol use during pregnancy (eight or more alcoholic drinks per week), known chromosomal or congenital anomalies potentially affecting the central nervous system, Apgar score at 5 minutes of less than five, any requirement for positive pressure ventilation in the Neonatal Intensive Care Unit (NICU), inability to return for outpatient magnetic resonance imaging (MRI) or follow-up, and intrauterine growth restriction below the third percentile. Unexposed (control) infants and mothers were recruited using similar criteria with a 2:1 ratio of exposed to unexposed infants. This analysis excludes data collected from nonbiological families.

Data included in this paper were collected at the 0 to 1-month study visit from mothers who took opioids and mothers who did not take opioids during pregnancy between November 2019 and December 2023. Specifically, this analysis includes 254 biological mothers who provided data on their food insecurity during pregnancy and housing instability at the 0- to 1-month study visit.

### Measures

#### Food Insecurity

Mothers screened positive for food insecurity during pregnancy if they responded “often true” or “sometimes true” to either or both of the following statements: During pregnancy, (1) we worried whether our food would run out before we got money to buy more; (2) the food we bought just didn’t last and we didn’t have money to get more. The screening questions were included using the two-item Hunger Vital Sign^™^ [15] screener as recommended by the American Academy of Pediatrics and the Food Research and Action Center [16]. Women who screened positive for food insecurity were provided information about federal nutrition programs and local food resources such as food pantries by study team members.

#### Housing and Neighborhood Characteristics

Housing instability was defined as spending excessive amount of income or moving in the last 6 months. Specifically, at the 0- to 1-month study visit, participants were asked: (1) Do you spend more than half (greater than 50%) of the household monthly income on housing costs?; and (2) “Have you moved in the last 6 months? The first item was selected as a measure of economic stability, defined as severely housing cost burdened [7, 8]. Participants who responded yes to either item were considered to have housing instability.

Additionally, other specific self-reported housing and neighborhood-related questions included on the study forms during the 0- to 1-month study visit were:

- Which best describes where the baby lives (participants were asked to indicate rented, owned by you or someone in the household [this includes with a mortgage/loan], or occupied without payment of rent)
- People in my neighborhood help each other out (participants were asked to indicate Disagree/Strongly disagree/Agree/Strongly agree/Neither)
- I feel safe in my neighborhood (participants were asked to indicate Disagree/Strongly disagree/Agree/Strongly agree/Neither)

#### Sociodemographic and Medical Characteristics

Data for the following characteristics were collected using medical chart review: maternal age, race, marital status, education, public insurance, parity, diagnosis of depression or anxiety disorder, and number of prenatal care visits. For this study, we defined adequate prenatal care as three or more visits and initiation of care before the third trimester. In addition, mothers were asked about traumatic events they may have experienced, including emotional or physical abuse during pregnancy and adverse childhood events using the Adverse Childhood Experiences questionnaire [17].

### Data Analyses

The percentage of study participants who screened positive for food insecurity was computed overall and by subgroup. Chi-square tests were used to compare food insecurity by the sociodemographic characteristics shown in Table 1.

In addition, we fit a logistic regression model of food insecurity by opioid use, maternal age, white race, marital status, education, public insurance, parity, depression or anxiety disorder diagnosis, number of adverse childhood experiences, physical or emotional abuse during pregnancy, and housing instability. Multiple imputation with 10 iterations was used to address missing values for demographic characteristics before inclusion in the regression analysis. A Cochrane-Armitage trend test was used to examine whether there is a linear trend of the relationship between number of adverse childhood experiences and food insecurity. Chi-square tests were conducted to compare food insecurity by housing and neighborhood characteristics. Similar analyses were used to examine the relationship between food insecurity during pregnancy and receipt of adequate prenatal care. SAS version 9.3 [18] was used for all analyses.

## RESULTS

Of the 254 mothers in the sample, 159 (63%) took opioids during pregnancy. The maternal age distribution was < 25 years (N = 45; 18%), 25–29 years (N = 80; 32%), 30–34 years (N = 85; 34%), and ≥ 35 years (N = 42; 17%). The majority were white (N = 201; 80%), had public insurance (N = 216; 86%) and were not married (N = 195; 81%). Nearly a quarter (N = 60; 24%) were primiparous and about half (51%; N = 129) had been diagnosed with depression or anxiety disorder. Over two-thirds (N = 172; 68%) reported having housing instability. A substantial portion of the sample had experienced traumatic events with 40% (N = 102) reporting three or more adverse childhood experiences and 22% (N = 57) reporting physical or emotional abuse during their pregnancy.

Overall, 58 (23%) of the mothers in the study were food insecure during their pregnancies. Mothers who took opioids during pregnancy were significantly more likely to have food insecurity (28% vs. 14%; p = 0.007; Table 1). Food insecurity was also more common among mothers who had public insurance (25% vs. 8%; p = 0.027). Housing instability was also associated with higher prevalence of food insecurity (28% vs. 11%, p = 0.002). Finally, mothers who had experienced three or more adverse experiences in their childhoods were more likely to have food insecurity during pregnancy (37% vs. 17%; p < 0.001) as were those who experienced physical or emotional abuse during their pregnancy (44% vs. 17%; p < 0.001). No significant differences in food insecurity were found for the other sociodemographic characteristics examined.

**Figure 1:**
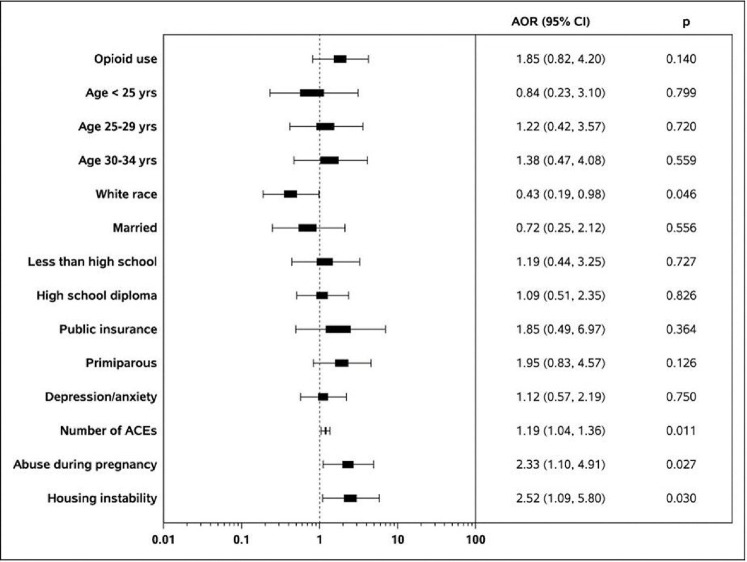
Adjusted Odds Ratios of Food Insecurity by Demographics, Drug Use, Housing, and Psychosocial Characteristics Note: ACE = adverse childhood experiences. Adjusted odds ratios control for opioid use, maternal age, white race, marital status, education, public insurance, parity, depression/anxiety, number of adverse childhood experiences, physical or emotional abuse during pregnancy, and housing instability.

After controlling for other factors, the difference in food insecurity based on opioid use was no longer significant (AOR (95% CI) = 1.85 (0.82, 4.20), p = 0.140) (Fig. 1).

In contrast to the unadjusted comparisons, racial differences in food insecurity emerged once controlling for other factors. Participants who were white had significant lower odds of experiencing food insecurity during pregnancy (AOR (95% CI) = 0.43 (0.19, 0.98), p = 0.046). Mothers with housing instability were also more likely to have food insecurity (AOR (95% CI) = 2.52 (1.09, 5.80), p = 0.030). Finally, mothers who had experienced trauma were more likely to be food insecure based on the number of adverse childhood experiences (AOR (95% CI) = 1.19 (1.04, 1.36), p = 0.011) and having experienced physical or emotional abuse during pregnancy (AOR (95% CI) = 2.33 (1.10, 4.91), p = 0.027).

Based on the regression results, we further examined the link between number of adverse childhood experiences and food insecurity. Overall, there is a general trend of greater percentages with food insecurity for more adverse childhood experiences (Cochrane-Armitage trend test: Z = 4.28, p < 0.001). Although 10%−13% of those with 0 or 1 adverse childhood experiences had food insecurity, 60% of those with 9 or 10 adverse childhood experiences were food insecure.

**Figure 2:**
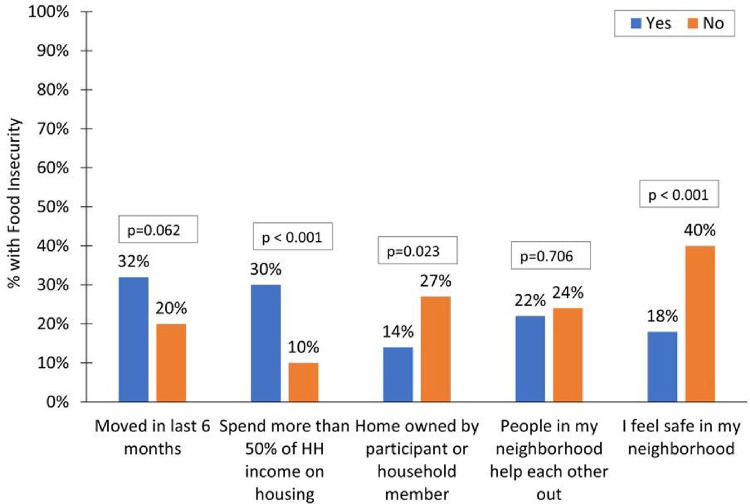
Food Insecurity by Household and Neighborhood Characteristics

Next, we explored further the relationship between housing characteristics and food insecurity given the regression results showing a positive association between housing instability and food insecurity. Of the two variables defining housing stability, only housing expenditures was significantly associated with food insecurity when examined separately; 30% of those who spent more than 50% of household income on housing were food insecure compared to 10% of those who did not (Fig. 2).

The relationship between moving in the last 6 months and food insecurity was not significant (p = 0.062). Among other housing-related factors, home ownership was associated with a smaller percentage having food insecurity (14% vs. 27%, p = 0.023). In addition, mothers who reported feeling safe in their neighborhoods were less likely to be food insecure (18% vs. 40%, p < 0.001).

Finally, we investigated whether food insecurity is associated with receiving adequate prenatal care. Mothers with food insecurity during pregnancy were less likely to have received adequate prenatal care (78% vs. 90%; p = 0.020). This difference remained after controlling for demographic characteristics (AOR (95% CI) = 0.39 (0.16, 1.00), p = 0.049).

## DISCUSSION

This study of at-risk mothers found twice the percentage with food insecurity among those who took opioids during pregnancy compared to those who did not take opioids. However, this difference was no longer significant when controlling for background characteristics. These findings suggest that the differences in food insecurity between the two groups may be attributable to other economic and sociodemographic factors beyond opioid use.

The study findings indicate that mothers who experience housing instability are at greater risk for food insecurity as well. Mothers with housing instability have 2.5 times the odds of having food insecurity. Among the two items used to assess housing instability, economic burden of housing costs, specifically spending more than 50% of the household income on housing, appeared to be the biggest driver of the relationship between housing instability and food insecurity, suggesting that rising housing costs may be particularly harmful to this at-risk population. Beyond housing instability, the results suggest that mothers who feel unsafe in their neighborhoods are more likely to have food insecurity, adding to the stress burden for mothers with concerns about securing food and housing.

Trauma appeared to be a strong risk factor for food insecurity. Even after controlling for sociodemographic factors and opioid use, mothers who reported physical or emotional abuse during pregnancy had more than two times the odds of experiencing food insecurity than those who did not report abuse. Trauma also had a lasting legacy with more adverse childhood experiences being associated with greater odds for food insecurity during pregnancy. The results from this study are consistent with research by Chilton and colleagues [19] who found that, among mothers with young children, those with four or more adverse childhood experiences were significantly more likely to have very low food security. The current study findings underscore the importance of a trauma-informed approach to care, a universal intervention that can be used provide care in the prenatal setting that recognizes the impact of trauma on health and employs supportive practices that actively avoid retraumatization and promote healing in individuals exposed to trauma [20, 21].

Food insecurity has been linked to poor pregnancy outcomes, including gestational diabetes, anemia, and pregnancy-induced hypertension [22], underscoring the importance of prenatal care to help mitigate these risks. Prenatal care also represents an opportunity for health care providers to screen for food insecurity and provide referrals to food assistance programs. However, in this study, we found an inverse relationship between food insecurity and receiving prenatal care. Despite the high levels of public insurance coverage among our sample (86% insured), mothers experiencing food insecurity were significantly less likely to receive adequate prenatal care, which is particularly concerning given the minimal number of prenatal care visits required to meet the study definition of adequate prenatal care. This finding suggests that perhaps another obstacle is preventing mothers from receiving prenatal care, such as transportation challenges [23] or stigma in health care settings encountered among women who have taken opioids during pregnancy [24].

Food insecurity and housing instability can have a particularly harmful effect on women during pregnancy as these social determinants of health do not exist in isolation. Unfortunately, the intersecting social determinants of health, such as the built environment, neighborhood safety, transportation options, literacy, and exposure to discrimination, can contribute to food insecurity and negatively affect the quality of postpartum life [25, 26]. A study conducted by Orr and colleagues highlights that mothers who experienced food insecurity attempted to follow infant feeding recommendations, but were less able than women with food security to sustain exclusive breastfeeding [27]. Our findings add to the body of evidence highlighting the relationship between housing and health [28], particularly among mothers of infants with antenatal opioid exposure for which there are limited data available. Our results amplify the call to action from others that regular screening during prenatal care visits to monitor the status of food- and housing-related issues should be explored [28]. Additionally, peer navigators or social workers who work with food- and housing-insecure pregnant and post-partum women can help them to identify and apply for existing services and resources within the community.

### Limitations

Data included in this analysis were limited to four clinical sites with strict adherence to robust inclusion/exclusion criteria so readers should be attentive to the generalizability of our results to the broader population of women who take opioids during pregnancy. For example, the infants enrolled in the OBOE Study were full term (≥ 37 weeks gestational age), in good general health following birth (i.e., did not require positive pressure ventilation in the NICU, had Apgar scores at 5 minutes of five or higher), with families expressing ability/willingness to return for outpatient MRIs and comprehensive follow-up visits.

## CONCLUSIONS

Our findings add to existing evidence highlighting the importance of food, housing, and health, particularly among mothers of infants with antenatal opioid exposure. Recognizing food insecurity and housing instability as key social determinants of health among this high-risk population suggests the need for screening during prenatal visits and referrals to available resources to improve outcomes and well-being.

## Figures and Tables

**Table 1:** Food Insecurity by Demographic Characteristics (N = 254)

Characteristic	Have Food Insecurity	
	n/N (%)	p
Opioid use		
Yes	45/159 (28)	0.007
No	13/95 (14)	
Maternal age		
<25	10/45 (22)	0.771
25–29	19/80 (24)	
30–34	21/85 (25)	
≥35	7/42 (17)	
White race		
Yes	43/201 (21)	0.287
No	15/53 (21)	
Marital status		
Married	6/47 (13)	0.060
Not married	50/195 (26)	
Education		
Less than high school	10/43 (23)	0.554
High school diploma	26/97 (27)	
More than high school	22/108 (20)	
Public insurance		
Yes	54/216 (25)	0.027
No	3/36 (8)	
Parity		
1	13/60 (22)	0.825
2+	44/191 (23)	
Depression/anxiety		
Yes	34/129 (26)	0.174
No	24/125 (19)	
Number of adverse childhood experiences		
0–2	20/152 (13)	<0.001
3+	38/102 (37)	
Physical and/or emotional abuse during pregnancy		
Yes	25/57 (44)	< 0.001
No	33/197 (17)	
Housing Instability		
Yes	49/172 (28)	0.002
No	9/82 (11)	

## Abbreviations

ACT NOW: Advancing Clinical Trials in Neonatal Opioid Withdrawal Syndrome
AOR: Adjusted odds ratio
CCHMC: Cincinnati Children’s Hospital Medical Center
CI: Confidence interval
MRI: Magnetic resonance imaging
NOWS: Neonatal opioid withdrawal syndrome
NICU: Neonatal Intensive Care Unit
OBOE: Outcomes of Babies with Opioid Exposure Study
OR: Odds ratio
RTI: Research Triangle Institute
